# 3D particle transport in multichannel microfluidic networks with rough surfaces

**DOI:** 10.1038/s41598-020-70728-1

**Published:** 2020-08-14

**Authors:** Duncan P. Ryan, Yu Chen, Phong Nguyen, Peter M. Goodwin, J. William Carey, Qinjun Kang, James H. Werner, Hari S. Viswanathan

**Affiliations:** 1 Center for Integrated Nanotechnologies, Los Alamos National Laboratory, Los Alamos, 87545 USA; 2 Computational Earth Sciences Group, Los Alamos National Laboratory, Los Alamos, 87545 USA; 3 Earth and Environmental Sciences, Los Alamos National Laboratory, Los Alamos, 87545 USA

**Keywords:** Energy science and technology, Fossil fuels, Computational science, Fluid dynamics, Techniques and instrumentation, Hydrology

## Abstract

The transport of particles and fluids through multichannel microfluidic networks is influenced by details of the channels. Because channels have micro-scale textures and macro-scale geometries, this transport can differ from the case of ideally smooth channels. Surfaces of real channels have irregular boundary conditions to which streamlines adapt and with which particle interact. In low-Reynolds number flows, particles may experience inertial forces that result in trans-streamline movement and the reorganization of particle distributions. Such transport is intrinsically 3D and an accurate measurement must capture movement in all directions. To measure the effects of non-ideal surface textures on particle transport through complex networks, we developed an extended field-of-view 3D macroscope for high-resolution tracking across large volumes ($25\,\hbox {mm} \times 25\,\hbox {mm} \times 2\,\hbox {mm}$) and investigated a model multichannel microfluidic network. A topographical profile of the microfluidic surfaces provided lattice Boltzmann simulations with a detailed feature map to precisely reconstruct the experimental environment. Particle distributions from simulations closely reproduced those observed experimentally and both measurements were sensitive to the effects of surface roughness. Under the conditions studied, inertial focusing organized large particles into an annular distribution that limited their transport throughout the network while small particles were transported uniformly to all regions.

## Introduction

Particles flowing through channels can be transported across streamlines due to inertial and collisional forces^1^. When particle size is comparable to the dimensions of a channel (within a couple orders of magnitude), movement can be strongly influenced by properties of and interactions with the local fluid flow as well as the topography of the channel surfaces. Many naturally occurring and artificial particle–fluid-surface systems operate at such size scales, and some applications even leverage these interactions to produce specific behavior. For example, flow cytometry can use specialized flow-cells to exploit inertial effects for passively sorting particles by size^2^. However, at such close proximity to channel walls, particle trajectories will also be impacted by the contours of those surfaces. In hydraulic fracturing for hydrocarbon extraction, proppants must be efficiently transported deep within fracture networks. Rough surfaces and complex fracture networks can restrict movement of these particles. Thus, developing comprehensive understanding of particle–fluid-surface interactions in this size regime is necessary for many fields.

Particle flow through channels has seen a great deal of interest in the microfluidics community as applications promise rapid and high-throughput particle processing^1–9^. For example, inertial focusing moves different sized particles to unique equilibrium positions within a stream and separation can be done by segmenting the fluid into bins by splitting the single channel among multiple outlets according to the equilibrium locations^1^. The majority of studies and applications focus on transport in 2D: networks confined within a single plane where channel depths are small compared to the other dimensions^4,5,10^. Multichannel separation only slices the stream along a single axis despite particles also distributing within the squeezed dimension. Particle tracking experiments to study these mechanisms of transport have largely been in 2D, which is experimentally simple to implement, requiring only imaging in 2D. However, such 2D treatment of a channel system is intrinsically not sensitive to surface roughness effects.

Although generally treated as ideal monolithic features, channels in real microfluidic devices have roughness from the manufacturing process. Similar surface roughness features occur in natural systems such as rock fractures. Microfluidics present an opportunity to create such systems in the laboratory. Near-surface effects due to roughness and shear flow near a boundary have been studied in flow-cells using nano-particle image velocity (nPIV) techniques^11–13^. These experiments demonstrate high-resolution 3D tracking but are limited in depth to sub micron-thick regions near channel surfaces where the imaging method is sensitive. Furthermore, to satisfy the total internal reflection angle condition for nPIV experiments, there are limits to the degree of roughness that can be explored.

Other 3D microscopy methods^14–21^ have been used to track particles within larger regions than are accessible with nPIV methods. Silva *et al.* used micro-PIV (fundamentally a different method than nPIV) to study the fluid velocity profile in a single microchannel with irregular walls to show deviation from the the theoretical parabolic flow velocity profile. However, this study did not interrogate the particle–fluid-surface interactions that mutually impact one another when particle size is large compared to the channel dimensions. A recent 3D tracking study using point spread engineering by Wang and Zhao examined the flow of moderate sized particles over a textured microcube array in a microchannel and found that particles did not follow steady state streamlines despite the constant flow rate^20^. While demonstrating the impact of particle–fluid-surface interactions on transport, the work relied on a periodic array that was imaged over a small field-of-view (FOV) to reduce the impact of edge effects due to texture changes. Examining large FOVs, such as an entire microfluidic device, in 3D with high enough resolution to be sensitive of subtle surface roughness has been difficult, in part because conventional microscopy tools are designed to reduce imaging distortions for smaller length scales and are not optimized for larger length scales. There is currently a measurement capabilities gap for 3D tracking at the larger length scales relevant to microfluidics ($10^{-2}$ to $10^{1}\hbox { mm}$) and new imaging techniques are necessary.

Despite advances in experimental techniques, exploring microfluidic devices with different patterns or under different flow conditions can still be challenging and time consuming. Some flow information such as the local pressure field cannot be easily determined from experiments. Numerical simulations, on the other hand, can provide detailed flow information and be rapidly adapted to different geometries and flow conditions. For high-fidelity results, simulations also require complementary experimental data to evaluate the validity or accuracy of such simulations. When particle size is comparable with the dimensions of a channel, the particles can no longer be treated as a point-like objects. Particle-resolved simulations are required in cases where the interactions between the solid surface of a moving particle and the surrounding fluid have to be modeled directly. The traditional finite element method^22^ can model the particle–fluid interactions with high accuracy but is very complex and computationally expensive due to the continuous regeneration of the body-fitting mesh. The immersed boundary method^23^ and the lattice Boltzmann method (LBM) with a particle-suspension model^24^ are among the most popular methods for simulations with fixed meshes. LBM is particularly suitable for low-Reynolds number flow in complex geometries, such as flow in porous media or flow in microfluidic devices with surface roughness, due to the efficient bounce-back non-slip boundary condition and high parallel performance on modern processors or accelerators^25–27^. In this work, we used an in-house developed LBM code^28,29^, which has been successfully employed in a similar microfluidic study^2^, to simulate the flow.

We present a 3D tracking macroscope to image extended FOVs compared to traditional 3D methods, and report on particle transport characteristics in a multichannel microfluidic network that exhibited particle–fluid-surface interactions. Channel surfaces were mapped with high-resolution profilometry to provide spatial context of experimental results and demonstrated the sensitivity of tracking measurements to roughness features. The surface profiles were also used in LBM simulation to build a precise model of the flow-cell network which led to high-fidelity matching of numerical and experimental results in a 3D environment. Exploring two particle sizes, we found inertial focusing organized larger particles into a distribution that did not propagate through all channels of the multichannel flow-cell while the smaller particles uniformly distributed into all of the channels. The organization and distribution behavior has acute implications for the effectiveness of particle sorting in flow cytometry and other microfluidic applications.

## Results

**Figure 1:**
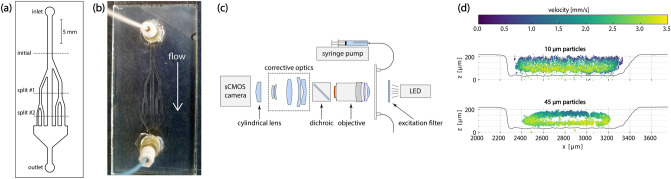
(**a**) Layout of the fracture network in the flow-cell. All channels were etched to a depth of ${\sim 200}\,{\upmu \hbox {m}}$, but have different widths. The initial segment of the flow cell was ${1000}\,{\upmu \hbox {m}}$ wide. At the location labeled *split #1*, the channels were ${500}\,{\upmu \hbox {m}}$, ${1000}\,{\upmu \hbox {m}}$, and ${750}\,{\upmu \hbox {m}}$ wide (left to right). The final channels, in the *split #2* region, were ${250}\,{\upmu \hbox {m}}$, ${500}\,{\upmu \hbox {m}}$, ${1000}\,{\upmu \hbox {m}}$, ${500}\,{\upmu \hbox {m}}$, and ${750}\,{\upmu \hbox {m}}$ wide (left to right). (**b**) Fully assembled flow-cell with inlet and outlet ports. The top surface in this photograph was the illuminated side and the bottom was the surface through which the flow-cell was imaged. The etched channels and inlet/outlet ports were components of the top plate while the bottom plate was a clean and smooth glass slide. (**c**) Schematic of the astigmatic macroscope used for 3D particle tracking. Fluorescent particles in the flow-cell are excited with a 470 nm LED fitted with a 477/60 nm emission filter. A microscope objective ($1.25\times$ or $2\times$ Plan Apo, Olympus) serves as the first element of a tandem lens pair with a 135 mm camera lens (Canon) serving as the second element of the pair and the corrective optics. A 488 nm dichroic beamsplitter in the infinity space between the pair is used to isolate the emission from the excitation. Astigmatism is introduced with a 400 mm focal length cylindrical lens before the camera. (**d**) ${10}\,{\upmu \hbox {m}}$ diameter (top) and ${45}\,{\upmu \hbox {m}}$ diameter (bottom) particle distributions measured at the position labeled *initial* in (**a**). Both distributions were measured at a volumetric flow rate of 25 $\upmu \hbox {L}/\hbox {min}$. The smaller particles are uniformly distributed within the channel at this point while the larger particles have formed an annulus.

A microfluidic flow-cell was used as a model multichannel network. Fluorescent polystyrene beads were used as tracking particles. Figure 1a shows the network layout: a single channel with multiple splits into channels of dissimilar dimensions. Cross-sectional particle distributions were measured at the locations labeled *initial*, *split #1,* and *split #2*. A fully assembled flow-cell is shown in Fig. 1b. We note that creating a channel network with multiple dissimilar sized channels was motivated by the solution of Wang *et al.* to use asymmetrical channels for improving cell sorting in flow cytometry^5^. Such network configurations are finding applications in microfluidic devices. The surface profile map of the network is shown in Fig. S2. To track particles moving through the network, an astigmatic macroscope, Fig. 1c, was designed to image the large FOV of the flow-cell (see “Methods”). Briefly, the imaging field of a standard, low-magnification microscopy objective was extended by pairing the objective with large aperture corrective optics. This configuration was necessary to eliminate aberrations produced in the large FOV (see Supplementary Fig. S1). To our knowledge, this is the first demonstration of such a corrective imaging system for particle tracking in this size regime. A cylindrical lens encoded the axial position (*z*) of the particles by introducing astigmatism into the image formation. An astigmatic image formation produces an elliptical spot that is oriented in one of two orthogonal directions depending on the location of the object above or below the focal plane. While this imaging configuration can track particles across the entire flow-cell, only particle distributions at the cross-sections indicated in Fig. 1a are presented in later discussion sections.

### Initial particle distributions

Figure 1d shows particle distributions measured in the region of the flow-cell, at the position labeled *initial*, immediately before the first channel split. The *z*-axis corresponds to the axial direction of the macroscope (the encoded third dimension). Particles measured in this region have settled into equilibrium positions following their entrance into the flow-cell. Upstream measurements indicated similar distributions for both particle sizes. At this point in the network, the ${10}\,{\upmu \hbox {m}}$ particles are distributed uniformly within the single channel. Roughness on the bottom surface produced during the flow-cell etching was mirrored in the distribution of the particles as an uneven, wave-like pattern in lower boundary. In contrast to the uniform distribution of the smaller particles, the larger ${45}\,{\upmu \hbox {m}}$ particles have formed a ring-like annular distribution by this position in the flow-cell. Furthermore, the edges of the distribution are smooth and do not reflect the surface profile of the flow-cell. Both particles sizes exhibit an asymmetric velocity profile in this region. The top surface of the flow-cell, where the velocities are slower, is a smooth glass surface while the bottom surface is the etched glass.

### Small particle transport

**Figure 2:**
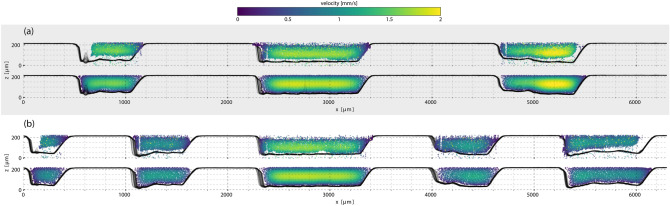
Cross sections of the particle distributions for ${10}\,{\upmu \hbox {m}}$ beads at a volumetric flow rate of 25 $\upmu \hbox {L}/\hbox {min}$. (**a**) Experimentally measured cross sections (top) and LBM simulations (bottom) after the first set of channel splits. (**b**) Experiment/simulation cross section pair after the second set of channel splits. The channel profiles within the region that the cross-section was measured (1 mm slice) are plotted as multiple overlapping semi-transparent black lines to indicate the local boundaries. Particle velocities are indicated by color. In both regions, the distribution edges follow the smooth top surface and irregular bottom surfaces of the flow-cell and were uniformly distributed within the channels. Consistent with Poiseuille (pressure-induced) flow, the highest particle velocities occurred in the center of the channels. The velocities decreased as the outer two channels split and the same volume of fluid expanded to fill the larger cross-sectional areas, unlike the center channel that did not split and had the same profile in both regions.

As particles flowed through the splitting segments and into the multichannel regions of the microfluidic device, the initial distributions in Fig. 1d were altered according to the layout of the network. Figure 2 shows the distributions of the ${10}\,{\upmu \hbox {m}}$ particles after the first channel splits (Fig. 2a) and after the second channel splits (Fig. 2b). The top plot of each panel displays experimental tracking results and the bottom plot of each panel shows the same region from LBM simulations. Simulations for the ${10}\,{\upmu \hbox {m}}$ particles were seeded by a uniform particle distribution because the initial distributions in Fig. 1d indicate such a distribution accurately describes the initial condition. Experimentally observed distributions and results from LBM simulations are consistent with one another. Several key features are reproduced in simulations that are a direct result of implementing the detailed surface profile of the flow-cell. The contours of the rough bottom surfaces are reflected in the cross-sections: the particle distribution envelopes show the same asymmetric profiles as the channels. For example, the right-most channel in Fig. 2a has a deeper distal region and a shallower medial region, which is reflected in the ballooning of the particles into the deeper segment of the channel in both experiment and simulation. The same sort of distribution reshaping to match channel cross-sections appears in the other channels as well. In contrast to the distributions across the rough lower surfaces, the upper edge of the particle distributions were flat, mirroring the smooth surface that forms the upper boundary (unetched glass slide).

In addition to matching particle density distributions, the simulations also well-match the measured particle velocity profiles. The only free parameter for the simulations was the flow-rate into the flow-cell (set by the programmable syringe pump). While the velocity distributions generally resemble the flow profile of a Poiseuille flow, due to the irregular surfaces and the uneven volume distribution of the fluid into multiple channels, particle velocities deviate from the theoretical flow profile. For example, the variable channel depths of the right-most channel in Fig. 2a produced a region of higher particle velocity that is asymmetrically positioned within the channel. Because the central channel is undisturbed by additional channel splits, particle velocities in this channel remain unchanged between the two measurement locations. However, the divisions of outer channels into multiple channels between Fig. 2a and b reduced particle velocities. LBM simulations captured the correct redistribution of fluid mass among the channels and resulted in reproducing the same velocity profiles as were experimentally observed.

### Large particle transport

**Figure 3:**
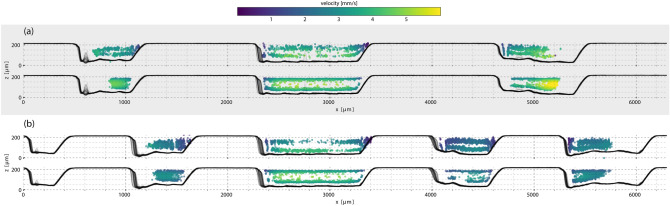
Cross sections of the particle distributions for ${45}\,{\upmu \hbox {m}}$ beads measured at a volumetric flow rate of 75 $\upmu \hbox {L}/\hbox {min}$. The upper plot of (**a**) shows the experimentally measured cross sections after the first set of channel splits and the lower plot shows the results from simulation. Similarly, the upper and lower plots in (**b**) show the experimental and simulation results, respectively, after the second set of channel splits. The distributions of ${45}\,{\upmu \hbox {m}}$ particles within the channels are not uniform: an annulus with no particles flowing through the centers of the channels. None of the ${45}\,{\upmu \hbox {m}}$ particles were transported into the smallest (bottom left) channel.

When the initial particle distribution in a network is not uniform, such as the annular distribution formed by ${45}\,{\upmu \hbox {m}}$ particles in Fig. 1d, the subsequent distributions after channel division further accentuate the heterogeneity. Figure 3 shows the experimental and simulation tracking results of the larger particles through the same network segments as discussed above. To inform the LBM model of the non-uniform initial particle distribution, the experimentally measured distribution in Fig. 1d was used to seed the simulations.

The initial annular distribution of the ${45}\,{\upmu \hbox {m}}$ particles is segmented by the channel divisions as the particles moved through the multichannel network. No significant reorganization occurred, although the annulus was deformed according to the irregular channel depths and roughly adopted the contours of the channels. As a consequence of the particle distribution occupying a smaller cross-sectional area of the initial channel, the extents of the ${45}\,{\upmu \hbox {m}}$ particle distribution were smaller. In Fig. 3b, only half of the right-most channel carries particles and the left-most channel contains none. This feature demonstrates a mechanism by which particle transport efficiency is reduced: not all channels within a network may be accessible due to non-uniform particle distributions. It is this feature by which flow cytometry separates particles. However, this also demonstrates how such sorting is prone to mixing. While all particles of a single experiment were the same size, segmenting by channels results in capturing a fraction of the same particle size in each channel.

## Discussion

Particle transport through the multichannel network was largely determined by the initial particle distributions. Channel splitting, surface roughness, and the flow parameters of this particular network did not produce conditions that could significantly disturb the initial distributions for either particle size. This behavior is characteristic of low-Reynolds number laminar flows where particle transport will primarily follow fluid streamlines. The Reynolds number for a particle in a channel is defined as^3^

1 $$\begin{aligned} \text {Re}=\dfrac{U_m a^2}{\nu D_h} \end{aligned}$$

where $U_m$ is the maximum velocity in the channel, $\nu$ is the kinematic viscosity of the fluid (the ratio of its dynamic viscosity, $\mu$, to its density, $\rho$), *a* is the diameter of the particle, and $D_h$ is the hydraulic diameter of the channel. For a rectangular cross-section channel of width *w* and height *h*, the hydraulic diameter is given by $D_h=2wh/(w+h)$. In this study, we determined dynamic viscosity of the brine to be $\mu =1.14\times 10^{-3}\,\hbox {Ns}/\hbox {m}^{2}$ and the density to be $\rho =1.05\times 10^{3}\,\hbox {kg}/\hbox {m}^{3}$ based on measured salt and water weights of the brine. This results in Reynolds numbers of $\text {Re}_{10\,\upmu \mathrm{m}}=0.002$ and $\text {Re}_{45\,\upmu \hbox {m}}=0.035$ for the ${10}\,{\upmu \hbox {m}}$ and ${45}\,{\upmu \hbox {m}}$ particles, respectively, and both particle species were within the laminar flow regime ($\text {Re}\ll 1$).

LBM simulations captured the evolution of the initial particle distributions though the flow-cell, matching experimental tracking measurements well. However, two discrepancies arose that were rooted in the degree of knowledge we have about the initial particle distributions within the channel. The first, illustrated in Fig. 2, is that experimental measurements do not show the ${10}\,{\upmu \hbox {m}}$ particle distributions extending completely to the outer edges of the most distal channels whereas simulations do. This is due to a uniform distribution being used for simulation whereas the true distribution was not exactly uniform. The initial distribution of the ${10}\,{\upmu \hbox {m}}$ particles, Fig. 1d, did not occupy the entire channel. While seeding simulations with an experimentally determined near-uniform distribution would correct this discrepancy, as was necessary for the ${45}\,{\upmu \hbox {m}}$ simulations, this solution also presents difficulties related to the second discrepancy: determining the exact location of the initial distribution. Figure 3a illustrates how the incorrect placement of an initial distribution due to incomplete information can result in differences between simulation and experiment. When the distribution is correctly positioned, such as Fig. 3b, the agreement is improved. However, repeated trials have shown experimental conditions can change in minor ways between measurements, for example air bubbles altering streamlines, and establishing the exact location of a distribution can be challenging.

**Figure 4:**
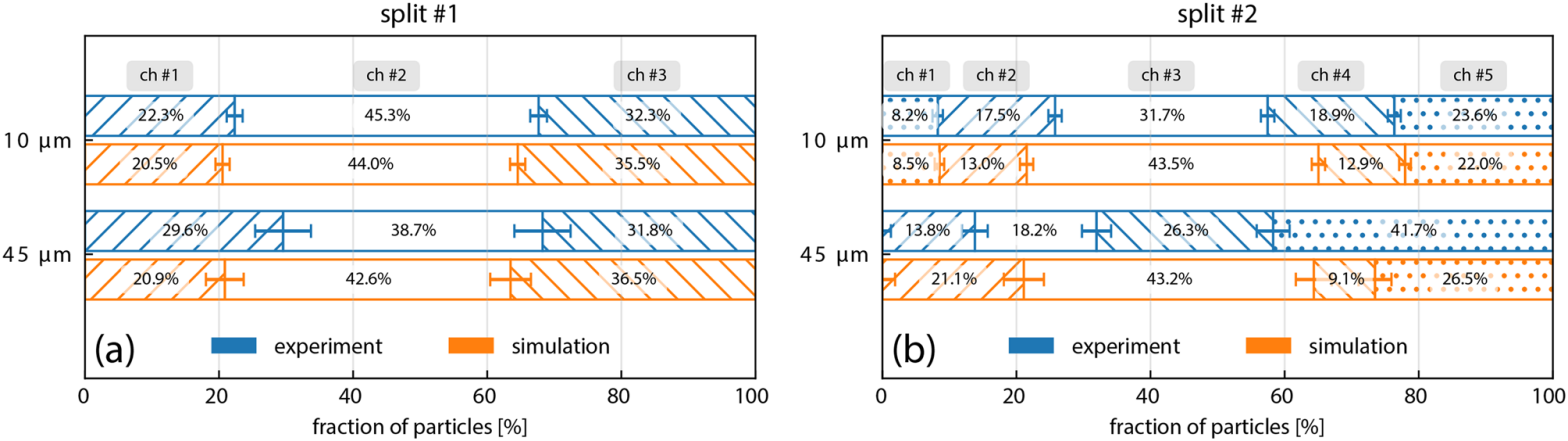
Ratios of particles distributed into each channel. (**a**) The relative numbers of particles distributed into each of the three channels after the first branching section of the flow-cell. The upper pair depicts the distributions for the ${10}\,{\upmu \hbox {m}}$ particles and the lower pair depicts the distributions for the ${45}\,{\upmu \hbox {m}}$ particles. Experimentally observed distributions are indicated in blue and the corresponding LBM simulations in orange. The central channel corresponds to the non-hatched segment while the outer channels are depicted with hatched bars. Uncertainty bars for the ratios correspond to $3\sigma$ of a multinomial distribution based on the number of particles in the ratio calculation. (**b**) The relative numbers of particles distributed into each of the five channels after the second branching section of the flow-cell. The outer-most channels are depicted with spot filling. Because none of the ${45}\,{\upmu \hbox {m}}$ particles entered the left-most channel, this segment does not appear in the lower pair. In all but the case of ${45}\,{\upmu \hbox {m}}$ particles after the second branching, the experimental and simulation results showed similar distributions.

The relative number of particles distributed into each channel provides a quantitative comparison of simulations and experiments. Figure 4 shows the ratios of particles that pass into each of the channels. After the first channel splitting, Fig. 4a, the ratios of both ${10}\,{\upmu \hbox {m}}$ and ${45}\,{\upmu \hbox {m}}$ particles are similar between the simulations and the experimental results. Due to the issue of precisely locating the center of the ${45}\,{\upmu \hbox {m}}$ particle initial distribution for seeding the simulations, there is a difference among the numbers of particles between the outer channels. The uniform initial distribution of the ${10}\,{\upmu \hbox {m}}$ particles resulted in nearly equivalent ratios. This close matching among the results extends to ratios determined after the second channel split, Fig. 4b. However, the ratios of ${45}\,{\upmu \hbox {m}}$ particles in this region differ more substantially. Most notably, the number of ${45}\,{\upmu \hbox {m}}$ particles flowing through the center channel (non-hatched segment) is smaller than were passing through the same channel after the first split. This is unexpected as no changes to the center channel occur after the first split (see the layout in Fig. 1a). Because the experimental measurements of each channel were separate experiments (imaged sequentially rather than simultaneously) it is possible that resetting and refilling of the syringe pump with the brine altered the flow. For example, the introduction of an air bubble could have been undetectable and pushed particles away from the center of the distribution.

**Figure 5:**
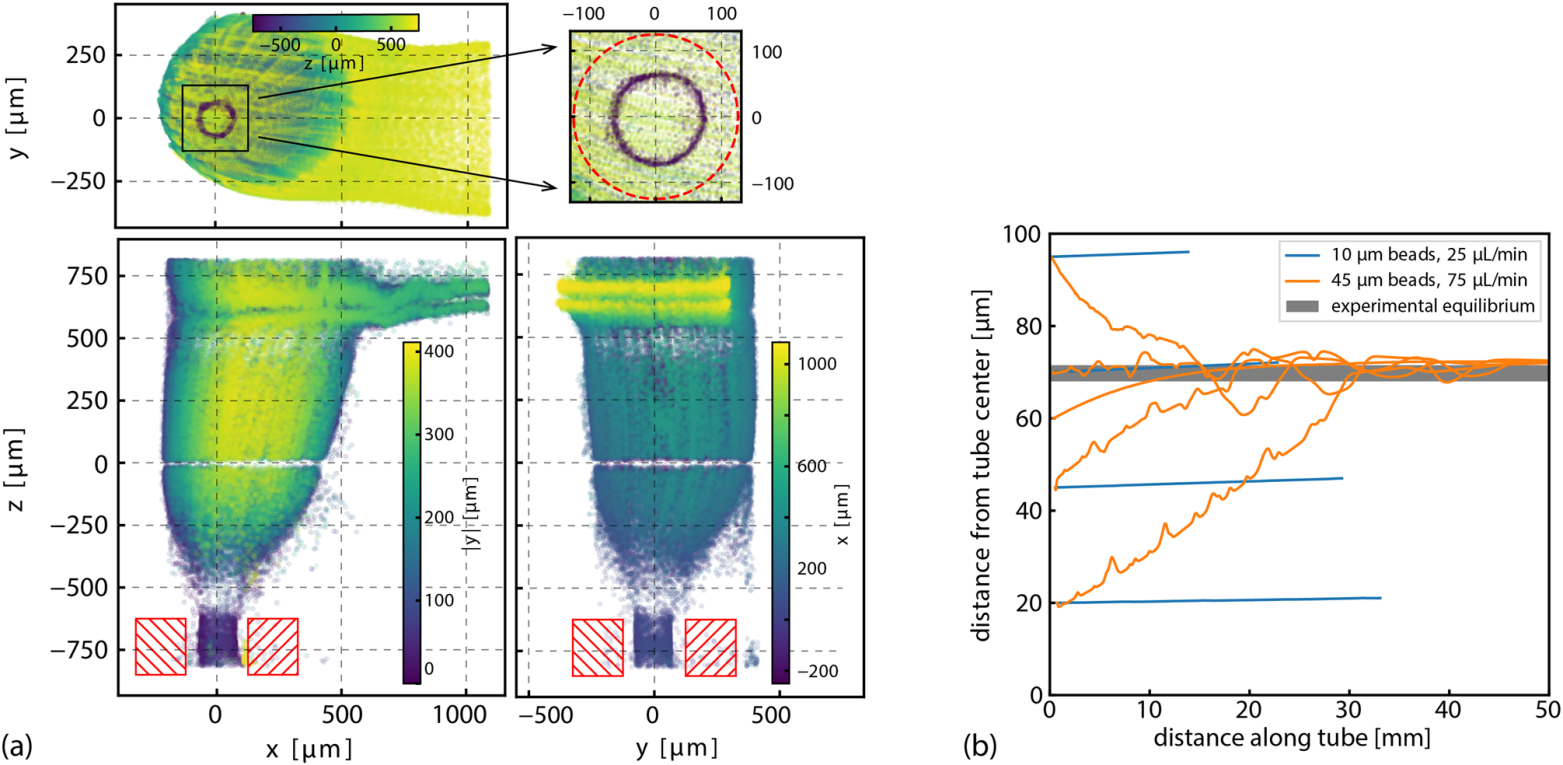
Inertial focusing of ${45}\,{\upmu \hbox {m}}$ particles from the feed tube. Orthogonal projections of the flow-cell inlet region at a volumetric flow rate of 25 $\upmu \hbox {L}/\hbox {min}$ are shown in (**a**) with a 1.25$\times$ objective for extended depth imaging. Color scales of each plot indicate distance along the flattened axis of each projection. The size and approximate position of the feed tube is indicated in red (hatched boxes and dashed circle). The distribution of the particles exiting the feed tube was an annulus smaller than the diameter of the feed tube. Expansion into the inlet region and compression into the rectangular cross-section of the initial channel is depicted in this figure. LBM simulations of the inertial focusing as the particles travel through the feed tube, (**b**), indicate the ${10}\,{\upmu \hbox {m}}$ particles did not migrate significantly while the ${45}\,{\upmu \hbox {m}}$ particles found an equilibrium at the same radial distance as the experimentally observed annulus. Tracks of several tracer particles starting at various radial distances from the center of the feed tube are shown. The length of feed tube of the experiment was $100\,\hbox {mm}$.

To investigate the mechanism that produced the different distributions between the two particle sizes, we examined the distributions upstream of the flow-cell. The distributions are already established before the initial segment of the network. Because of the construction of the microfluidic flow-cell, fluid and particles enter through an inlet region that is composed of a feed tube and an antechamber that accommodates the coupling of the feed tube into the initial channel. In this region, the flow takes a 90$^{\circ }$ turn, expanding into the larger volume of the antechamber before being compressing into the smaller cross-sectional area of the initial channel. Figure 5 show trajectories of ${45}\,{\upmu \hbox {m}}$ particles entering this region where the streamlines undergo this complex reshaping. As previous works have shown, eddies can form under such conditions^30^. There is evidence, most notably in the *xz*-projection, that particle movement here is effected by such streamlines. However, eddies do not appear to substantially disrupt the larger non-uniform particle distribution entering the flow-cell. In Fig. 5a, the size of the ${250}\,{\upmu \hbox {m}}$ diameter feed tube is outlined in red. The magnified insert (upper right) shows particles exiting the feed tube in an annular distribution with a diameter that is 54 % of the feed tube’s diameter. ${10}\,{\upmu \hbox {m}}$ particles exit the feed tube in a uniform distribution. Thus, before the larger particles have reached the flow-cell, they have been sorted into a non-uniform distribution. The 90$^{\circ }$ direction change does not substantially disrupt this initial distribution within the low-Reynolds number regime and the same annular shape appears in at the end of the initial single channel before the splitting has occurred.

The role of the feed tube in producing different distributions for the ${10}\,{\upmu \hbox {m}}$ and ${45}\,{\upmu \hbox {m}}$ particles can be explored using LBM simulations of particles moving through an ideal circular tube. Figure 5b shows the migration of tracer particles from various radially distributed starting locations. After ${30}\,\hbox {mm}$ the ${45}\,{\upmu \hbox {m}}$ particles have coalesced to the same radial position within the circular tube while the ${10}\,{\upmu \hbox {m}}$ particles have not migrated any significant distance from their initial radial positions. Furthermore, the equilibrium location of the larger particles matches the diameter of the annular distribution experimentally observed in Fig. 5a. This migration of the tracer particles is due to inertial focusing transporting particles across streamlines^1,4,5,31–33^. In flows with a velocity gradient, such as in Poiseuille flow, the different fluid velocities on opposite surfaces of a particle produce a net force that moves the particle until an equilibrium position is obtained^2^. Large particles experience greater fluid velocity differentials across their diameters than smaller particles.

## Conclusions

We demonstrated a 3D tracking macroscopy method that images particle transport across large FOVs. This approach is particularly useful for microfluidic applications where typical flow-cell dimensions can be difficult to image with traditional methods. Interactions among particles, fluid, and their microchannel environment, such as the rough contours of channel surfaces, were investigated using a 3D macroscopy approach. We observed small ${10}\,{\upmu \hbox {m}}$ particles in low-Reynolds number flows transported through a multichannel flow-cell in a uniform distribution and their spatial distributions reflected the rough contours of the channel walls. Such close tracking of these particles with the channel surfaces indicate they would be ideal for infiltrating small features in complex networks because of their proximity to branching points. However, larger ${45}\,{\upmu \hbox {m}}$ particles experienced inertial focusing before entering the flow-cell. In the main cavity of the flow-cell, changes to these distributions were dominated by channel splitting. As a result of the non-uniform initial distribution, the larger particles were not transported into all channels. These experiments demonstrated why the purity of size sorting in microfluidics is limited. Particles are distributed within the cross-sectional areas of the channels and segmentation along a single direction will combine particles across different depths. 3D imaging demonstrates this behavior that is not evident in 2D experiments.

Direct comparison of fluid dynamic experiments with simulations often requires simplifying model complexity and can produce mixed results. Reducing the treatment of a system to 2D or ignoring detailed boundary conditions from textured surfaces has limited the convergence of these approaches. However, we have shown that particle transport can be accurately modeled using LBM and that results closely match experimentally observed behavior. Demonstrated on a simplified microfluidic system, such an approach as presented here can be expanded to more complex systems. For example, particle transport in random fracture patterns with extreme surface textures—more closely resembling naturally occurring systems—could be explored using this microfluidic approach.

## Methods

### 3D astigmatic macroscope

The axial position (depth) of a single particle is encoded into the shape of the image it produces in astigmatic imaging. Typical high-magnification microscopes can image a particle within only a small volume, otherwise strong aberrations (such as vignetting and image distortion) occur that defeat the shape encoding used to determine the axial position. To track particles in 3D over extended fields of view, we introduce the astigmatic macroscope, which utilizes a set of corrective optical elements in a tandem lens configuration to reduce these aberrations. Measurements with the macroscope were made with a $2\times$ Plan Apo objective lens (Olympus) as the first element of the tandem pair and a 135 mm telephoto lens (Canon), focused at infinity, as the second element. A 400 mm focal length cylindrical lens between the telephoto lens and the sCMOS camera generated the astigmatism to encode axial positions. The resulting magnification of the system was $1.48\times$.

${10}\,{\upmu \hbox {m}}$ and ${45}\,{\upmu \hbox {m}}$ diameter Fluoresbrite YG microspheres (Polysciences, Inc.) were used as tracking particles. The microspheres were excited in a trans-illumination configuration with a 470 nm light emitting diode (LED). A 447/60 nm excitation filter (Semrock FF02-447/60) was used to clean up the LED spectral profile. Emission from the microspheres is peaked at 486 nm and a 488 nm dichroic beamsplitter (Semrock Di02-R488) located within the infinity space of the tandem pair isolated the microsphere emission from the excitation light.

Given the imaging conditions in this work, the axial uncertainties were ${16.5}\,{\upmu \hbox {m}}$ and ${4.4}\,{\upmu \hbox {m}}$ for the ${10}\,{\upmu \hbox {m}}$ and ${45}\,{\upmu \hbox {m}}$ microspheres, respectively. The corresponding lateral uncertainties were 530 nm and 100 nm. The difference between the uncertainties is primarily due to the fact that the larger beads contain more dye and are brighter.

### Microfluidic flow-cell

The multichannel network was comprised of a single $1000\,{\upmu \hbox {m}} \times 200\,{\upmu \hbox {m}}$ initial channel that splits into three channels, followed by a second set of channel splits that result in five channels at the end of the flow-cell (see Fig. 1a). Each branching segment splits the set of channels into additional channels of dissimilar widths. This network was etched into a 3 mm quartz slide using a programmable $\hbox {CO}_{2}$ laser (Gravograph LS100)^34,35^. Power fluctuations during the etching and overlap of the raster pattern produced uneven and rough etched surfaces. The surface profile of the flow-cell (see Supplementary Fig. S2) was mapped with an optical-interference profilometer (Keyence VK-X100). This profile was used to define the channel boundaries for simulations and provides a feature overlay of experimental results.

The flow-cell was enclosed by adhering an unetched glass slide to the fracture slide using a low viscosity UV-curing glue (Norland NOA133). Because of the low viscosity, the glue wicked between the surfaces where the slides made direct contact but did not expand into the etched channels, eliminating potential gaps that could form and would have produced additional, unintended paths for fluid flow. Inlet and outlet ports were drilled perpendicular to the fracture flow direction, creating 90$^{\circ }$ direction changes as the fluid and particles entered and exited the flow-cell. The fully assembled flow-cell with inlet/outlet ports is shown in Fig. 1b.

### Measurement and analysis

The enclosed flow-cell was connected to a syringe pump and a brine solution containing the fluorescent particles was pumped through the flow-cell at a constant volumetric flow-rate. To negate the effects of gravity, the water density was increased to match that of the polystyrene beads, requiring 8 % w/w NaCl in $\hbox {H}_{2}\hbox {O}$. The brine also contained 0.025 % Tween 20, a surfactant, to reduce particle adhesion to the to the flow-cell surfaces. Either ${10}\,{\upmu \hbox {m}}$ beads or ${45}\,{\upmu \hbox {m}}$ beads were diluted into a brine solution from their stock solutions to obtain low particle concentrations appropriate for singe-particle imaging ($\sim 10^{3}\,\hbox {particles}/\upmu \hbox {L}$).

For cross-sectional distributions of particles within the individual channels, only segments of the entire FOV needed to be imaged. Imaging ${275}\,{\upmu \hbox {m}}$ long slices along the direction of flow provided multiple localizations of individual particles, from which velocity measurements could be extracted, while also reducing the overall storage space and processing time required for a measurement. The reduced data footprint allowed for longer measurements to be easily taken that produced more complete distribution data. To prevent blurring as the particles moved, images were taken with 5 ms exposures at 50 ms intervals. Two flow rates were used: 25 $\upmu \hbox {L}/\hbox {min}$ for the ${10}\,{\upmu \hbox {m}}$ particles and 75 $\upmu \hbox {L}/\hbox {min}$ for the ${45}\,{\upmu \hbox {m}}$ particles. We found that, within this range, the flow-rates did not noticeably affect the distributions of either particle size.

Particle positions were determined from the raw images using ThunderSTORM, an analysis package for single-molecule localization microscopy^36^. Determining particle trajectories (and velocities) requires linking localizations across frames. We implemented a simple tracking algorithm that correlates particle localizations using a probability cost-matrix based on the assumption of linear flow and additional constraints specific to the experimental configuration. For additional details, see the Supplementary Information.

### LBM simulations

We used an LBM code developed in-house that has been successfully employed to simulate particle inertial focusing in microfluidic devices^2^. The main variable in LBM is the discretized particle distribution function $f_i$, and the governing equations with the popular BGK collision model^37,38^ are,

2 $$\begin{aligned} f_i(\varvec{x}+\varvec{e}_i\delta t,t+\delta t)=f_i(\varvec{x},t)-\frac{f_i(\varvec{x},t)-f_{i}^{eq}(\varvec{x},t)}{\tau }, \end{aligned}$$

where $f_i$ is the particle distribution function associated with the *i*-th discrete velocity direction $\varvec{e}_i$, $f_{i}^{eq}$ is the corresponding equilibrium distribution function, $\delta t$ is the time increment, and $\tau$ is the relaxation time. The relaxation time relates to the kinematic viscosity by

3 $$\begin{aligned} \nu =(\tau -1/2)c_s^2\delta t, \end{aligned}$$

where $c_s$ is the speed of sound. We employed the three-dimensional and nineteen-speed D3Q19 lattice model^37^ to simulate 3D flow.

Applying non-slip boundary conditions on complex geometries is challenging for traditional computational fluid dynamics, such as the case of flow in porous media or microfluidic devices with roughness. One of the main advantages of LBM is the bounce-back nonslip boundary scheme^25^ where the nonslip condition can be completed by reversing the directions of the distribution functions on the solid boundary nodes. Here, we applied the bounce-back scheme on the rough channel walls. For the moving particles with limited grid resolution, we employed a curved boundary condition^39^ to the solid surfaces of the particles to model particle–fluid interactions with sub-grid resolution. We adopted the corrected momentum-exchange method^28^ to evaluate the hydrodynamics forces exerted on the particles. This method ensures relatively smooth force transitions as particles move across lattice nodes and avoids complex and inefficient schemes such as the stress-integration method^40^. The motion of a particle is then obtained by solving Newton’s equations^41^:

4 $$\begin{aligned} M\frac{d\varvec{U}(t)}{dt}=\varvec{F} \end{aligned}$$

and

5 $$\begin{aligned} \varvec{I}\cdot \frac{d\varvec{\Omega }(t)}{dt}+\varvec{\Omega }(t)\times [\varvec{I}\cdot \varvec{\Omega }(t)]=\varvec{T}, \end{aligned}$$

where *M*, $\varvec{I}$, $\varvec{U}$, and $\varvec{\Omega }$ are the mass, rotational inertia, velocity, and angular velocity of the particle, respectively; $\varvec{F}$ and $\varvec{T}$ are the force and torque exerted on the particle, respectively. A more detailed description of the numerical methods can be found in our previous works^28,29^.

Simulations involving the entire microfluidic device used a grid resolution of ${7.5}\,{\upmu \hbox {m}}$, balancing computation time with simulation resolution, which results in a $932\times 29\times 2592$ computational grid. The mapped regions of the flow-cell did not include the inlet or outlet regions. Simulations directly resolving particle–fluid interactions were done for the ${45}\,{\upmu \hbox {m}}$ particles. Because the large particles established a non-uniform distribution before the initial single-channel segment of the flow-cell, experimentally obtained distributions were used to seed the simulations. The evolution of the distribution as the particles moved through the network was captured with this approach.

Modifications to the computation scheme were necessary for the ${10}\,{\upmu \hbox {m}}$ particles. Because the smaller particles were approximately the same size as the grid, particle-resolved simulations were too coarse to directly incorporate their interactions with the fluid using the momentum-exchange method. LBM simulations of inertial focusing in the feed tube for both particles sizes were possible because of the reduced complexity of the boundaries and smaller computational demands, and one can significantly increases the grid resolution to study ${10}\,{\upmu \hbox {m}}$ particle transport in the feed tube. As demonstrated by these simulations, shown in Fig. 5b, ${10}\,{\upmu \hbox {m}}$ particles do not show significant inertial migration at these flow rates and channel dimensions because the ratio between the diameter of the particle and the aperture of the channels is relatively large. As a result, a point-particle tracking method with one-way coupling could be employed for full chip simulations of the ${10}\,{\upmu \hbox {m}}$ particles. In such an approach, the particles are treated as volumeless objects that do not affect the flow field, and only the Stokes’ drag $\varvec{F}_d$, computed from the relative velocity of the particles and surrounding fluid, is considered to update particle positions. In this case, the drag force has the form

6 $$\begin{aligned} \varvec{F}_d=6\pi \mu r \varvec{V}_{relative}, \end{aligned}$$

where $\mu$ is the dynamic viscosity of the fluid, *r* is the particle radius, and $\varvec{V}_{relative}$ is the relative velocity between the particle and surrounding fluid.

## Supplementary information

Supplementary Information.
