# Supplementary material for: 3D particle transport in multichannel microfluidic networks with rough surfaces
Source: Sci Rep. 2020 Aug 14;10:13848. doi: 10.1038/s41598-020-70728-1 (PMC7427810; doi:10.1038/s41598-020-70728-1)
Supplement: Supplementary file 1 — Supplementary Information. [file 41598_2020_70728_MOESM1_ESM.pdf]

Supplementary Information for: 3D Particle Transport in Multichannel Microfluidic Networks with Rough Surfaces

Duncan P. Ryan[1], Yu Chen[2], Phong Nguyen[3], Peter M. Goodwin[1], J. William Carey[3], Qinjun Kang[2], James H. Werner[1], and Hari S. Viswanathan[3,*]

[1]Center for Integrated Nanotechnologies, Los Alamos National Laboratory, Los Alamos, 87545, USA
[2]Computational Earth Sciences Group, Los Alamos National Laboratory, Los Alamos, 87545, USA
[3]Earth & Environmental Sciences, Los Alamos National Laboratory, Los Alamos, 87545, USA
[*]viswana@lanl.gov

Aberration Correction

To track particles in 3D across a field-of-view (FOV) as large as 25 mm, we implemented a design based on a tandem lens optical system.[1] A tandem lens system is a pair of infinity focused compound lenses oriented such that the infinity-corrected side of one lens faces the infinity-corrected side of the other. By using a wide aperture photography lens as one element of the pair, the 3D astigmatic macroscope takes advantage of the aberration correction offered by compound lenses as well as the larger acceptance angle. The compound lens configuration of a photography lens is designed to form an image in the compressed (shortened) distance of a camera body, correctly focusing large angle rays at the imaging plane. Figure S1 compares the use of a typical tube lens, Figs. S1(a–c), versus a compound photography lens, Fig. S1(c). Two imaging problems arise when using a simple tube lens. The first is vignetting (Fig. S1(a)) where the light collection efficiency across the field of view is not uniform. This has the disadvantage of limiting the field-of-view (FOV) over which imaging is possible as well as reduced localization precision for particles near the edges of the FOV because they appear dimmer. The second imaging issue is aberration due to the extreme angles some rays exit the microscope objective. This manifests as a point-spread function (PSF) whose shape is dependent on where it is located within the FOV. Figure S1(b) shows this effect in detail. Near the edges of the image, the PSFs appear rotated and do not have the same size as PSFs located near the center of the image. Because the axial dimension is encoded into the shape of the PSF in astigmatic imaging, this aberration alters the image such that extracting 3D information is not possible. Finally, Fig. S1(c) shows the imaging quality with the compound lens instead of the tube lens. Over the same FOV, no vignetting is present and the PSFs are not distorted.

Tracking algorithm

Raw image data is processed into fully connected particle tracks for tracking visualization and to extract velocity information. We used the software package ThunderSTORM to determine the three position coordinates of every spot in an image sequence, generating a list of time-indexed localizations (x_i, y_i, z_i, t_i). While several tracking methods have been published to collect individual localizations into tracks of the same particle,[2] we found it more reliable to use a custom algorithm that took advantage of some assumptions that apply to our measurements.

The algorithm generates a cost-matrix by calculating the probability a given localization is the same particle as another localization given their separation distance and the number of frames between the localizations. A threshold is set to group localizations belonging to the same particle and the cost-matrix is searched for localization groups that have high probabilities of being sequential localizations of the same particle. For cross-section measurements we assume that flow is primarily in one direction and that the maximum velocity, set by the limits of the imaging area, constrains the distances where the particle would be found in subsequent frames. Thus, for the cost-matrix elements comparing the i-th localization to the $(i+j)$-th localization, the probability function has the form:

$$P\left(x_{i+j}, y_{i+j}, t_{i+j}, x_i, y_i, t_i\right) = P^{\text{Gauss.}}\left(x_{i+j} - x_i|\sigma_x\right) \times \int P^{\text{Gauss.}}\left(v_y \times (t_{i+j} - t_i) - (y_{i+j} - y_i)\,|\sigma_y\right) \times P^{\text{Uni.}}\left(v_y\right) dv_y \tag{1}$$

where $P^{\text{Gauss.}}$ and $P^{\text{Uni.}}$ are normal and uniform probability distributions, respectively. The integration is over the range $[0, v_{y_{\max}}]$, limited by the extents of the image area and imaging interval. The widths of the distributions, σ_x and σ_y, function as tolerance

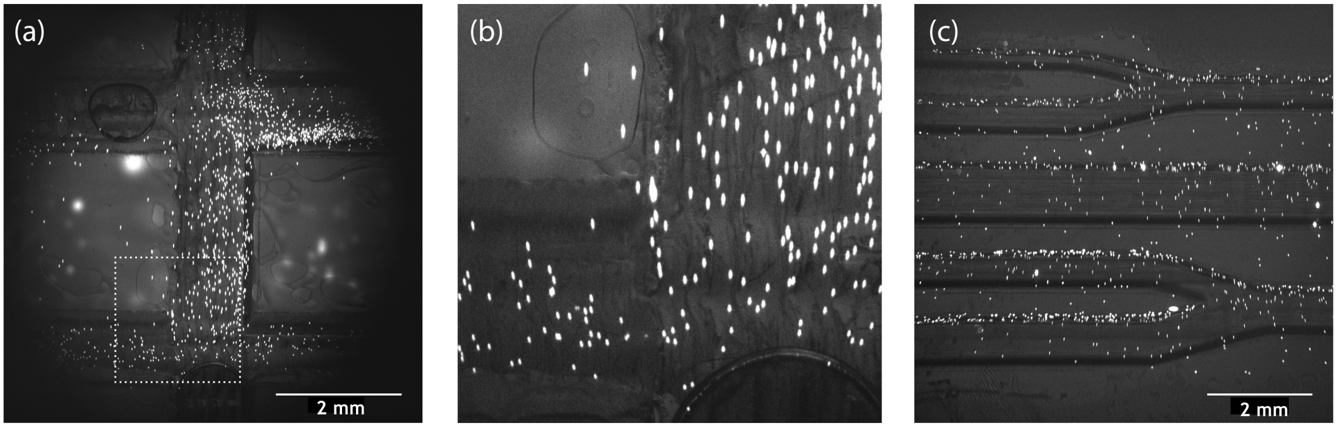

Figure S1. Imaging aberration that occur without corrective optics. **(a)** Full FOV image produced when a 180 mm tube lens is used instead of the corrective optics in Fig. **??**(b). Vignetting produces a shadow region near the edges of the image. **(b)** A close-up of the boxed region in (a). PSFs produced by single particles are distorted and appear to be dependent on their locations radially as well as azimuthally within the image. Farther away from the image center, PSFs incur a rotation due to aberrations. **(c)** The same FOV image with the astigmatic 3D macroscope and the compound lens. The imaging aberrations in (a) and (b) are no longer present. Scale bars in (a) and (c) are 2 mm.

parameters for motion in the given direction. Furthermore, the cost-matrix is constrained by the requirements $y_{i+j} > y_i$ and $t_{i+j} > t_i$. These requirements enforce forward motion in the y-direction and time. This probability function only considers the motions in the lateral directions, x and y. While the axial information could be incorporated, it was not necessary for the measurements in this study because movement in the z-direction was slow and the particle density was kept low enough that multiple particles at similar xy-positions were not common. The application of this probability model for tracking particles is shown in pseudocode in Algorithm 1.

Algorithm 1 Tracking algorithm

Given $\theta = \{(x_1, y_1, z_1, t_1), \ldots, (x_N, y_N, z_N, t_N)\}$
Initialize $\Phi = \{\}$
for $i < N$ **do**
  if $\theta_i \notin \Phi$ **then** ▷ Localization does not belong to another track.
    $\phi = \{\theta_i\}$
    for $j = 0$ to $N - i$ **do**
      if $t_{i+j} > t_i$ **and** $y_{i+j} > y_i$ **then** ▷ Forward motion in y.
        $\alpha = P(x_{i+j}, y_{i+j}, t_{i+j}, x_i, y_i, t_i)$ ▷ Calculate probability.
        if $\alpha \geq \alpha_{\text{thres.}}$ **then**
          $\phi \leftarrow \{\theta_{i+j}\} \cup \phi$ ▷ Add localization to track.
        end if
      end if
    end for
    $\Phi \leftarrow \phi \cup \Phi$ ▷ Add track to list of all tracks.
  end if
end for

Here, θ is the set of all localizations, comprised of their spatial and temporal coordinates, N is the total number of localization over all frames of an image sequence, $\alpha_{\text{thresh.}}$ is a thresholding parameter for the minimum probability to connect two localizations, and Φ is the final list of all tracks where each element ϕ contains the individual localizations θ_i that belong to an individual track. Because this algorithm effectively calculates the cost-matrix for many unlikely localization pairs, it is not particularly efficient. However, it has demonstrated low false-positive and false-negative rates for this tracking problem.

Flow-cell Device

A complete profilometry map of the microfluidic flow-cell used in this work is shown in Fig. S2. Here, the multiple channel splits and the final reservoir are seen. In the profilometry map, strips of slightly different depths were present in the final reservoir region and in individual channels. These were due to laser power fluctuations within the raster scan during etching and are responsible for the surface roughness. The uniformly distributed 10 μm particles closely tracked these surface features and the roughness was reflected in their distributions.

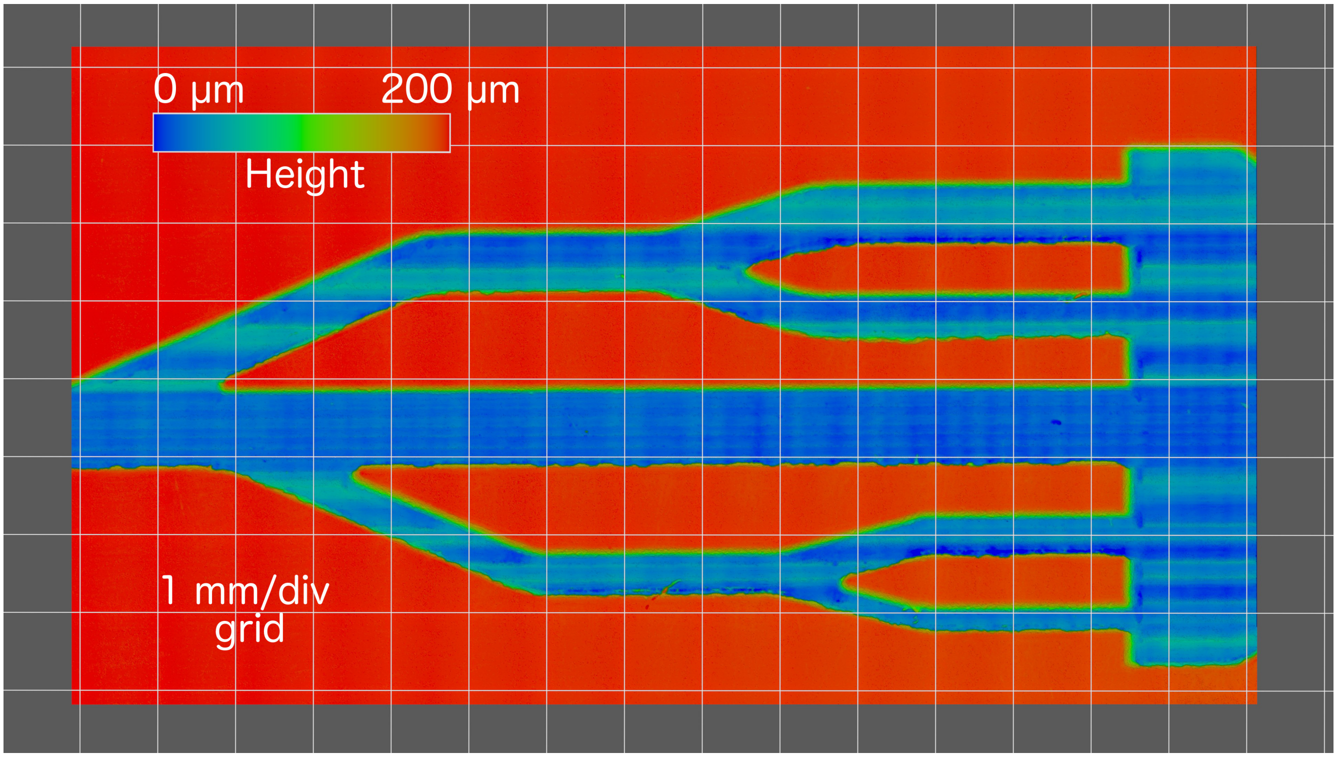

Figure S2. Profilometry characterization of the flow-cell showing the topology of the etched channels.

References

1. Ratzlaff, E. H. & Grinvald, A. A tandem-lens epifluorescence macroscope: Hundred-fold brightness advantage for wide-field imaging. *J. Neurosci. Methods* **36**, 127–137, DOI: 10.1016/0165-0270(91)90038-2 (1991).

2. Tinevez, J.-Y. *et al.* TrackMate: An open and extensible platform for single-particle tracking. *Methods (San Diego, Calif.)* **115**, 80–90, DOI: 10.1016/j.ymeth.2016.09.016 (2017).
